# The Synergistic Effects of Jasmonic Acid and Arbuscular Mycorrhizal Fungi in Enhancing the Herbicide Resistance of an Invasive Weed *Sphagneticola trilobata*

**DOI:** 10.3390/microorganisms13122817

**Published:** 2025-12-10

**Authors:** Hu’anhe Xiong, Misbah Naz, Rui Chen, Mengting Yan, Zongzhi Gong, Zhixiang Shu, Ruike Zhang, Guangqian Ren, Shanshan Qi, Zhicong Dai, Daolin Du

**Affiliations:** 1 Institute of Environment and Ecology, School of the Environment and Safety Engineering (Key Laboratory of Zhenjiang), Jiangsu University, Zhenjiang 212013, China; xhah0518@163.com (H.X.); misbahnaz.ray@yahoo.com (M.N.); yanmengting0412@163.com (M.Y.); 18355451587@163.com (Z.G.); zhangruike0601@163.com (R.Z.); rgq@ujs.edu.cn (G.R.); 2 State Key Laboratory of Green Pesticide and Agricultural Bioengineering, Guizhou University, Guiyang 550025, China; 3 School of Agricultural Engineering, Jiangsu University, Zhenjiang 212013, China; chenrui2212416002@163.com (R.C.); szx2496308234@163.com (Z.S.); qishanshan1986120@163.com (S.Q.); 4 Jiangsu Collaborative Innovation Center of Technology and Material of Water Treatment, Suzhou University of Science and Technology, Suzhou 215009, China; 5 Jingjiang College, Jiangsu University, Zhenjiang 212028, China; 6 School of Emergency Management, Jiangsu University, Zhenjiang 212013, China

**Keywords:** arbuscular mycorrhizal, herbicides resistance, invasive weed, phytohormone, plant-microbial interaction

## Abstract

The invasive plant *Sphagneticola trilobata* (Asteraceae), known for its rapid growth and strong adaptability, has spread widely across tropical and subtropical regions worldwide, posing a serious threat to local plant diversity. Traditional weed control approaches have limited effectiveness, and the overuse of chemical herbicides such as glyphosate not only leads to resistance but also harms the environment. This study elucidated the important roles of jasmonic acid (JA) and arbuscular mycorrhizal fungi (AMF) in jointly promoting the herbicide resistance of *S. trilobata*. Firstly, the herbicide tolerance of *S. trilobata* was tested. Then, a field experiment was conducted to test the relation between AMF colonization and herbicide resistance in *S. trilobata* by high-throughput sequencing, and the metabolomics analysis was conducted to test the secondary metabolite difference by AMF colonization. Lastly, a greenhouse experiment was conducted to assess the synergistic effects of JA and AMF on *S. trilobata*’s herbicide resistance. Results showed that invasive *S. trilobata* has stronger glyphosate tolerance than its native congener. The field experiment showed that glyphosate treatment significantly increased the AMF colonization in *S. trilobata* and altered the composition of the rhizosphere AMF community. Metabolomics analysis revealed that AMF colonization upregulates the expression of stress-related metabolites, especially JA content. The greenhouse experiment further validated that both AMF colonization and JA application could enhance the stem and root length and plant biomass. Under glyphosate application, AMF and JA enhanced plant growth and relative chlorophyll content, while reducing relative flavonol and anthocyanin contents. Furthermore, the interaction of AMF and JA treatments led to a significant synergistic effect in plant growth and resistance to glyphosate. Our findings emphasize the necessity to simultaneously consider eliminating the promoting effects of JA and AMF on the herbicide resistance when implementing chemical control management strategies for the control of *S. trilobata*. This study provides new theoretical bases and sustainable control strategies for invasive plant management, as well as important references for research on plant-microbe interactions and stress resistance mechanisms.

## 1. Introduction

The spread of invasive plant species poses a significant threat to biodiversity, ecosystem stability, and agricultural productivity [1,2]. Invasive plants typically grow rapidly and are highly resistant to stress conditions, enabling them to quickly monopolize resources and form dominant populations [3,4]. They reproduce in a variety of ways, spread rapidly, and are highly tolerant to environmental changes and pests [5,6]. These characteristics make invasive plants extremely difficult to eradicate completely, and conventional physical or chemical control approaches are often ineffective [7,8]. Therefore, their management has become a long-term challenge in ecological protection, requiring sustained and comprehensive management strategies. One such invasive species is *Sphagneticola trilobata* (L.) Pruski (Asteraceae), which has spread across tropical and subtropical regions, outcompeting native flora and disrupting ecosystems [9]. *S. trilobata* is an herbaceous perennial plant native to Central and South America but is now widespread in many tropical and subtropical regions, including Southeast Asia, Africa, and the Pacific Islands. Its invasiveness is attributed to several factors, including its ability to form dense ground cover that suppresses native plant species, its capacity for vegetative reproduction, and its tolerance to a wide range of environmental conditions [10]. These traits make *S. trilobata* a formidable competitor in both disturbed and undisturbed ecosystems. Once established, this species alters nutrient cycling, reduces biodiversity, and can affect the structure and function of ecosystems. Controlling *S. trilobata* has been a significant challenge. However, traditional mechanical and chemical control methods, including manual removal and the application of herbicides like glyphosate, have been only partially successful [11]. The extensive use of herbicides to manage invasive species has raised concerns about the development of herbicide resistance in target plants [12]. Herbicide resistance can occur through various mechanisms, including changes in the herbicide target site, enhanced metabolic detoxification of the herbicide, or alterations in herbicide absorption and translocation within the plant [13]. Known for its rapid vegetative growth, high reproductive rate, and adaptability to various environmental conditions, *S. trilobata* has become difficult to manage using conventional methods, including the application of herbicides [14].

Glyphosate is widely employed for the control of invasive species, but its overuse can lead to the development of resistance, negative environmental impacts, and non-target species damage [15,16]. It is a broad-spectrum herbicide and acts by inhibiting the shikimate pathway, essential for the biosynthesis of aromatic amino acids in plants [17]. However, over-reliance on glyphosate has led to several issues, including resistance in certain plant populations and adverse environmental effects, such as soil degradation and the contamination of water bodies [18]. Glyphosate resistance in various species has become an increasingly significant issue, particularly in invasive plants that possess high genetic variability and adaptability. In the case of *S. trilobata*, there is evidence suggesting that this species may possess traits that allow it to tolerate glyphosate treatment; when it comes to its native species, *S. calendulacea* (L.) Pruski (Asteraceae), few studies report on their tolerance to glyphosate. This tolerance may arise from physiological adaptations, such as increased production of antioxidants to mitigate oxidative stress, or changes in root morphology that limit herbicide uptake [19,20]. Consequently, managing *S. trilobata* through herbicide application alone is becoming increasingly unsustainable, necessitating the exploration of alternative strategies that enhance plant resistance while reducing reliance on chemicals [19]. In a field survey, we found that herbicide does not completely kill *S. trilobata*, but that new leaves could regrow from the wilting stems and new shoots from the roots (Figure S1). Hence, the mechanisms that confer strong resistance to herbicides of this invasive weed need to be well studied.

Arbuscular mycorrhizal fungi (AMF) are symbiotic organisms that form mutualistic associations with the roots of most terrestrial plants [21]. Through this symbiotic relationship, AMF enhance plant nutrient acquisition, particularly phosphorus, and improve water uptake, which is critical for plant survival under stress conditions. In return, the plant supplies the fungi with carbohydrates derived from photosynthesis [22]. AMF are known to confer numerous benefits to their host plants, including enhanced tolerance to drought, salinity, and heavy metal toxicity. Recent studies have also demonstrated that AMF can improve plant resistance to herbicides [23]. By improving nutrient and water uptake, AMF-colonized plants are better able to withstand the physiological stress induced by herbicides [24]. Additionally, AMF can influence plant defense signaling pathways, including the JA pathway, thereby enhancing the plant’s overall stress response. AMF colonization has been shown to alter root morphology, increasing root surface area and promoting root branching, which may reduce herbicide absorption and improve plant resilience [23,25]. Furthermore, AMF can enhance the production of antioxidant compounds in plants, which helps to detoxify reactive oxygen species (ROS) generated during herbicide exposure [26].

Jasmonic acid (JA) has a potential role in enhancing plant herbicide resistance, and we should focus on its synergistic effects in mitigating herbicide-induced stress [27]. JA is a plant hormone that plays a central role in regulating plant responses to biotic and abiotic stresses [28]. Initially identified for its role in plant defense against herbivores, JA has since been recognized for its broader functions in modulating plant growth, development, and stress responses. The JA signaling pathway is activated in response to various environmental stimuli, including wounding, pathogen attack, and herbicide exposure [29]. Upon activation, JA triggers the expression of defense-related genes that lead to the production of secondary metabolites, such as flavonoids, alkaloids, and terpenoids, which protect plants from damage [30]. JA also interacts with other plant hormones, such as salicylic acid (SA) and ethylene, to fine-tune defense responses based on the type of stress encountered [31]. For example, while SA is typically involved in defense against biotrophic pathogens, JA is more closely associated with responses to necrotrophic pathogens and herbivores [32]. This hormone crosstalk allows plants to allocate resources efficiently, optimizing defense mechanisms in response to specific threats. Several studies have highlighted JA’s potential role in enhancing plant resistance to herbicides [33]. By regulating the production of detoxifying enzymes and antioxidant compounds, JA can mitigate the oxidative damage caused by herbicides like glyphosate. Furthermore, JA has been shown to influence root architecture, improving the plant’s ability to absorb water and nutrients under stress conditions [34]. Hence, we hypothesize that JA may contribute to enhancing the herbicide resistance of invasive species like *S. trilobata*.

There is growing evidence that JA and AMF interact synergistically to enhance plant stress tolerance [35,36,37,38,39]. The crosstalk between JA signaling and AMF colonization may lead to a more robust defense response in plants, as AMF can enhance JA-mediated defense pathways [40]. However, does the interaction between JA and AMF symbiosis contribute to the herbicide resistance of invasive weeds? To address this gap, we conducted a comprehensive study to (1) evaluate the herbicide tolerance of the invasive *S. trilobata*, (2) analyze the changes in AMF communities after herbicide application to the invasive weed in field, (3) detect the JA content of AMF symbiosis with the invasive weed, and (4) investigate the interaction of JA and AMF in the herbicide resistance of the invasive weed in a greenhouse experiment. This study could give a clear explanation about the contribution of JA and AMF to the herbicide resistance of invasive weeds.

## 2. Materials and Methods

### 2.1. Test of Plant Tolerance to Herbicide

To detect the herbicide tolerance of the invasive *S. trilobata*, four different concentrations of 77.7% glyphosate ammonium salt soluble granule herbicide (Jiangsu Good Harvest-Weien Agrochemicals Co., Ltd., Qidong, Jiangsu, China) were applied to *S. trilobata* and its native congener *S. calendulacea* according to previous studies [41,42]. Stems of *S. trilobata* were collected from Huizhou City (23°12′ N, 113°96′ E), Guangdong Province, China, and stems of *S. calendulacea* were collected from a greenhouse at Jiangsu University (32°12.02′ N, 119°31.76′ E), Zhenjiang City, Jiangsu Province, China; both were propagated in the greenhouse at Jiangsu University. The glyphosate concentrations included 0, 50, 100, and 200 mg·L^−1^ for both species, with 5 replicates per treatment (4 treatment × 5 replicates × 2 species = 40 plants). Plant stem segments were inserted into plastic flower pots (9 ×8 ×8 cm), which were filled with 250 g of washed and dried river sand. After 2 weeks of plant growth, herbicide solutions of different concentrations were sprayed onto the plant leaves and irrigated into the roots every 2 weeks for herbicide treatment. Each pot received 10 mL of spray and irrigation. The control group (herbicide concentration of 0 mg·L^−1^) received the same volume of sterile water sprayed onto the leaves and irrigated into the roots. All flower pots were randomly placed in the greenhouse (temperature: 25 °C; photoperiod: 16 h/8 h) for cultivation. To ensure plant growth, sterile water was applied daily, and 0.5 × Hoagland nutrient solution was added weekly to meet the plants’ nutritional requirements.

After about two months of growth, all the plants were harvested. After herbicide application, some leaves became brown and withered, so we counted these damaged leaves in each plant. The tolerance index of each part of the plant is calculated using the following formula [43]:

$$Tolerance\;index=\frac{\;Biomass_{herbicide\;application}\;}{Biomass_{Without\;herbicide\;application\;}}$$

### 2.2. Field Sampling for AMF Diversity Response to Herbicide

To explore the AMF community response to herbicides, a field experiment was carried out in a wasteland of Guangxi Botanical Garden of Medical Plants (22°51.2′ N, 108°22.27′ E), Nanning, Guangxi Province, according to the method by Hu et al., 2024 [44]. Two populations of *S. trilobata* with the same growth stage were randomly selected. In each population, there were three plots (2 m × 2 m) that were about 20 m apart. One population received no herbicide application (CK), while the other population was treated with 800 mg·L^−1^ of 77.7% glyphosate ammonium salt soluble granule herbicide (HC) every month for four times. After approximately half a year of plant growth, 8 random plant individuals from the 3 plots in each population were collected (2 treatment × 8 replicates = 16 plants), and fine roots were sampled from a depth of 10–30 cm below the soil surface around each plant for AMF colonization detection. Rhizosphere soil was also collected and was homogenized, passed through a 2 mm sieve, and then stored at −20 °C for later determination of AMF diversity.

### 2.3. Assessment of AMF Colonization

According to the method of Qi et al. [45], hyphal staining was performed, and the AMF colonization rate was assessed. The fine roots were removed, washed with sterilized water, and cut into root segments approximately 2 cm long. First, the root segments were digested in a 10% KOH solution at 70 °C for 7 min, then rinsed with distilled water; the root segments were then rinsed in a 30% H_2_O_2_ solution for 5 min, followed by rinsing with distilled water; subsequently, the root segments were acidified in a 1% HCl solution for 3 min, then rinsed with distilled water. Next, place the root segments in a 0.05% trypan blue solution and stain them in a 70 °C water bath for 15 min, then remove and rinse with sterilized water. Finally, place the rinsed root segments in a 50% lactic acid solution for decolorization for 10 min, remove them, and observe the mycelium morphology under a microscope. The mycelium abundance of the infected root segments is classified into the following four grades: 0–25%, 26–50%, 51–75%, and 76–100%. The mycorrhizal infection rate is calculated using the following formula [46]:

$$Mycorrhizal\;colonization\;rate\left(\%\right)=\frac{Number\;of\;infected\;root\;segments}{Total\;number\;of\;roots\;segments}\times 100\%\;$$

### 2.4. Analysis of AMF Diversity

The rhizosphere soil stored at −20 °C was mixed uniformly and transferred into pre-labeled 2 mL centrifuge tubes for high-throughput sequencing analysis of AMF at Beijing Biomarker Technologies Co., Ltd. (Beijing, China). DNA from AMF in the rhizosphere soil was extracted using the TGuide S96 magnetic bead-based soil genomic DNA extraction kit (Tiangen Biotech (Beijng) Co., Ltd., Beijing, China). The concentration of the extracted DNA was measured using a microplate reader (FlexA−200, Hangzhou Allsheng Instruments Co., Ltd., Hangzhou, China). Based on the measurement results, the DNA was amplified. The PCR products were subjected to electrophoresis using 1.8% agarose to assess their integrity. After passing the quality control, the DNA was used to construct a library, which was subjected to quality control using the Qsep-400 method. Fusion primer PCR was performed using total DNA from each sample as a template. The PCR amplification primers were specific primers for AMF, AMV4.5NF (5’-AAGCTCGTAGTTGAATTTCG-3’), and AMDGR (5’-CCCAACTATCCCTATTAATCAT-3’) [47]. PCR system (10 μL): 5–50 ng of sample genomic DNA, 0.3 μL each of primers AMV4.5NF (10 μmol·L^−1^) and AMDGR (10 μmol·L^−1^), 5 μL of KOD FX Neo Buffer, 2 μL of dNTP (2 mmol·L^−1^ each), 0.2 μL of KOD FX Neo, and _dd_H_2_O to a total volume of 10 μL. PCR conditions: 95 °C for 5 min; 95 °C for 30 s, 50 °C for 30 s, 72 °C for 40 s, repeated for 25 cycles; 72 °C for 7 min; store at 4 °C.

The purity of the PCR amplification products was detected using 1.8% agarose gel electrophoresis. Column purification was performed using the DNA Purification Kit (Omega Bio-tek, Inc., Norcross, GA, USA). The purified products were used as templates for the second round of PCR amplification. The PCR products were mixed in equal amounts based on their concentrations and purified again. Gel recovery was performed using the Monarch DNA Gel Recovery Kit (New England Biolabs, Ipswich, MA, USA), and high-throughput sequencing was conducted on the Novaseq 6000 (Illumina, Inc., San Diego, CA, USA) sequencing platform. Sequencing was performed by Beijing Biomarker Technologies Co., Ltd. (Beijing, China), followed by assembly and quality filtering of the raw reads, and removal of chimeric sequences to obtain the final valid data, which were categorized into features (OTUs, ASVs), diversity analysis, and differential analysis. The Silva SSU reference database (v138) was used to taxonomically classify the ASV sequence with a naive Bayes classifier, and the community composition of each sample group was aggregated at the genus level. Sequences were clustered at a similarity level of 97%, with a threshold of 0.005% of the total number of sequenced sequences used to filter OTUs. First, the feature sequences were aligned with reference databases (Silva, Unite, Greengenes, NCBI, Fungene, MaarjAM) using classify-consensus-blast. Sequences that could not be precisely aligned with the reference databases were classified using the classify-sklearn classifier, and OTUs were annotated taxonomically. We used QIIME2 (v2020.6) to calculate Chao1 richness, ACE richness estimator, and Shannon diversity index from the rarefied OTU table. The R package vegan was used to perform a permutational multivariate analysis of variance (PERMANOVA) analysis.

### 2.5. Metabolomics Analysis of the Effect of AMF on the Growth of S. trilobata

To access the secondary metabolites, especially JA changes in the AMF symbiosis with *S. trilobata*. We planted *S. trilobata* stems inoculated with an AMF strain *Entrophospora etunicata* (W.N.Becker & Gerd.) Błaszk., Niezgoda, B.T.Goto & Magurno. The AMF inoculum used in this experiment was self-propagating, which contained about 14 spores per gram [48]. There are two treatments in this experiment, including AMF inoculation (+AMF) and non-AMF inoculation (−AMF). For +AMF treatment, the mixture of 10 g of *E. etunicata* and 240 g of sand was added to the flowerpots, and 10 g of sterile inoculum (sterilized at 121 °C for 2 h) mixed with 240 g of sand for the −AMF treatment. There were three replicates for these two treatments (2 treatments × 3 replicates = 6 plots). All the pots were placed in the greenhouse of Jiangsu University and watered daily with sterile water and supplemented with 1× Hoagland nutrient solution once a week. The leaf samples of these two treatments were collected for metabolomics analysis (Suzhou Panomix Biomedical Tech Co., Ltd., Suzhou, China) after 2 months of incubation.

Metabolites from the leaf samples were extracted according to the method described by Vasilev et al. [49]. Then we weighed appropriate amounts of the leaf samples and placed them in 2 mL centrifuge tubes, added 600 µL of methanol solution (containing 2-chloro-L-phenylalanine at 4 ppm), stored at −20 °C, vortexed for 30 s, then placed the centrifuge tube containing 100 mg of glass beads into a tissue grinder and ground at 60 Hz for 90 s. Next, sonicate at room temperature for 15 min, then centrifuge at 4 °C and 12,000 rpm for 10 min. Finally, filter the supernatant through a 0.22 μm membrane, add the filtrate to the detection vial, and use it for LC-MS detection.

The LC analysis was performed on a Vanquish UHPLC System (Thermo Fisher Scientific Inc., Waltham, MA, USA). Chromatography was carried out with an ACQUITY UPLC ^®^ HSS T3 (150 × 2.1 mm, 1.8 μm) (Waters, Milford, MA, USA). The column was maintained at 40 °C. The flow rate and injection volume were set at 0.25 mL/min and 2 μL, respectively. For LC-ESI (+)-MS analysis, the mobile phases consisted of (C) 0.1% formic acid in acetonitrile (*v*/*v*) and (D) 0.1% formic acid in water (*v*/*v*). Separation was conducted under the following gradient: 0~1 min, 2% C; 1~9 min, 2%~50% C; 9~12 min, 50%~98% C; 12~13.5 min, 98% C; 13.5~14 min, 98%~2% C; 14~20 min, 2% C. For LC-ESI (−)-MS analysis, the analytes were carried out with (A) acetonitrile and (B) ammonium formate (5 mM). Separation was conducted under the following gradient: 0~1 min, 2%A; 1~9 min, 2%~50%A; 9~12 min, 50%~98%A; 12~13.5 min, 98%A; 13.5~14 min, 98%~2%A; 14~17 min, 2%A.

Mass spectrometric detection of metabolites was performed on Orbitrap Exploris 120 (Thermo Fisher Scientific Inc., Waltham, MA, USA) with an ESI ion source. Simultaneous MS1 and MS/MS (Full MS-ddMS2 mode, data-dependent MS/MS) acquisition was used. The parameters were as follows: sheath gas pressure, 30 arb; aux gas flow, 10 arb; spray voltage, 3.50 kV and −2.50 kV for ESI(+) and ESI(−), respectively; capillary temperature, 325 °C; MS1 range, m/z 100–1000; MS1 resolving power, 60,000 FWHM; number of data dependent scans per cycle, 4; MS/MS resolving power, 15,000 FWHM; normalized collision energy, 30%; dynamic exclusion time, automatic.

We converted the raw mass spectrometry data files into mzXML format, used the R package XCMS to perform peak detection, peak filtering, and peak alignment on the data, obtaining a list of quantified compounds. Under parameter settings where the molecular weight error is less than 30 ppm, we used public databases such as HMDB (http://www.hmdb.ca, accessed on 1 December 2025), MassBank (http://www.massbank.jp/, accessed on 1 December 2025), LipidMaps (http://www.lipidmaps.org, accessed on 1 December 2025), mzCloud (https://www.mzcloud.org, accessed on 1 December 2025), KEGG (http://www.genome.jp/kegg/, accessed on 1 December 2025), and the in-house standard substance library (BioDeep, http://v2.biodeep.cn/, accessed on 1 December 2025) of Panomix Biomedical Tech Co., Ltd. (Suzhou, China) for substance identification. Finally, the R package Ropls was used to perform PCA, differential metabolite analysis, and JA content analysis.

### 2.6. Role of Jasmonate Acid and AMF in Enhancing Herbicide Resistance of S. trilobata

To validate the roles of JA and AMF in herbicide resistance of *S. trilobata*, a factorial block design greenhouse experiment was conducted. Plant stems were inserted into plastic flower pots (9 × 8 × 8 cm), which were filled with 250 g of washed and dried river sand. There were eight treatments in this experiment: herbicide treatment (+HC) with 77.7% ammonium glyphosate soluble granules, and without any herbicide application (−HC). In addition, there were four treatments in each herbicide treatment. That is, (1) control treatment (CK), where there was no AMF inoculation or JA application. (2) AMF inoculation treatment (+AMF), where the mixture of 10 g of *E. etunicata* (approximately 140 ± 20 spores) and 240 g of sand was added to the flowerpots. (3) JA application treatment (+JA), where methyl dihydrojasmonate and jasmonic acid have similar signaling functions and can activate the same defense genes and metabolic pathways [50,51,52,53,54]. Therefore, plants were treated with 2 mg·L^−1^ methyl dihydrojasmonate (Sangon Biotech, Shanghai Co., Ltd., Shanghai, China) after one week of growth. (4) Plants received both AMF inoculation and JA treatment (+AMF + JA) during growth. For herbicide treatment, a 100 mg·L^−1^ herbicide solution was sprayed on the leaves of *S. trilobata* every two weeks, and the roots were irrigated after the new branches of *S. trilobata* grew to about 10 cm. An herbicide solution was sprayed twice during the experiment. There are five replicates for these eight treatments (8 treatments × 5 replicates = 40 plants). We randomly placed all the pots in the greenhouse under natural light. In order to meet the nutritional requirements for plant growth, Hoagland’s nutrient solution was added every week [55].

After two months of growth, all forty plants were harvested, and growth variables were determined, including stem length, leaf biomass, stem biomass, root length, and root biomass. The relative content of chlorophyll, flavonol, and anthocyanin in the second pair of leaves from the top of each plant was determined by the Multi pigment measuring MPM-100 GPS instrument (Opti-Sciences Inc., Hudson, NH, USA). The WinRHIZO root analysis system (Regent Instrument Inc., Québec, QC, Canada) was used to scan the root morphology. Each part of the plant was dried at 80 °C to constant mass, and the biomass (dry weight) of the plant was obtained after weighing.

### 2.7. Data Analysis

Before data analysis, the normality and homogeneity of variance of all the data were assessed using IBM SPSS Statistics V24.0 software. In the field experiments, A t-test was used to evaluate the significant differences in mycorrhizal colonization rate and in each hyphae abundance range between CK and HC treatments. A *t*-test was also used to evaluate the significant differences in JA content between −AMF and +AMF treatments. In the greenhouse experiment, Tukey’s test (*p* < 0.05) was used to test the difference in growth and physiological indicators among different treatments, and the interactions of JA, AMF, and herbicide on the growth of *S. trilobata* were evaluated by a three-way ANOVA. All data were plotted using Origin 2021 (ver. 9.8.0.200) software.

## 3. Results

### 3.1. Plant Tolerance to Herbicide Concentration Gradients

**Figure 1:**
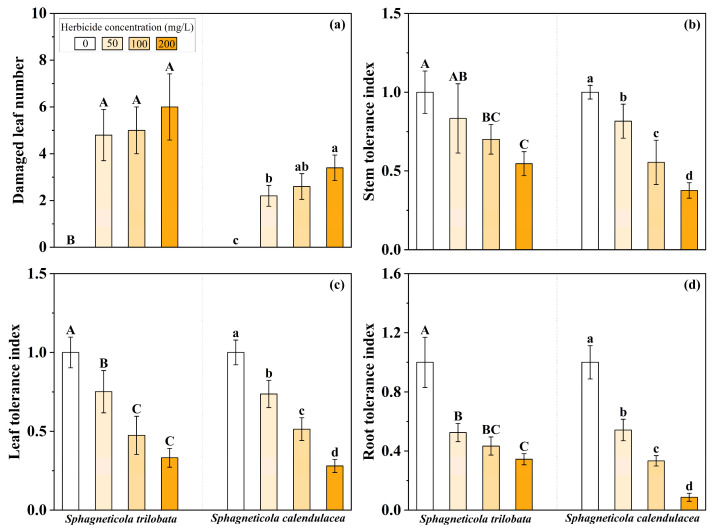
Damaged leaf number (**a**), stem tolerance index (**b**), leaf tolerance index (**c**), and root tolerance index (**d**) of invasive *Sphagneticola trilobata* and native *Sphagneticola calendulacea* under different concentrations of herbicide. Different letters indicate significant differences under different treatments. (*p* < 0.05, Mean ± SE, *n* = 5).

It was found that all three herbicide concentrations can cause leaf damage both for *S. trilobata* and *S. calendulacea,* with no significant difference for *S. trilobata* among the gradient herbicide concentrations. While the damaged leaf number applied with the highest concentration was significantly higher than with low concentration in *S. calendulacea* (Figure 1a). As herbicide concentration increases, the tolerance index of stem, leaf, and root of *S. trilobata* and *S. calendulacea* showed a significant decreasing trend (Figure 1b–d). Notably, there was no significant difference between 50 and 100 mg·L^−1^, or between 100 and 200 mg·L^−1^ in the stem and root tolerance index, and between 100 and 200 mg·L^−1^ in the leaf tolerance index for *S. trilobata*; however, there were significant differences among these gradient concentrations in *S. calendulacea*.

### 3.2. The Effects of Herbicide on the AMF Community of S. trilobata

**Figure 2:**
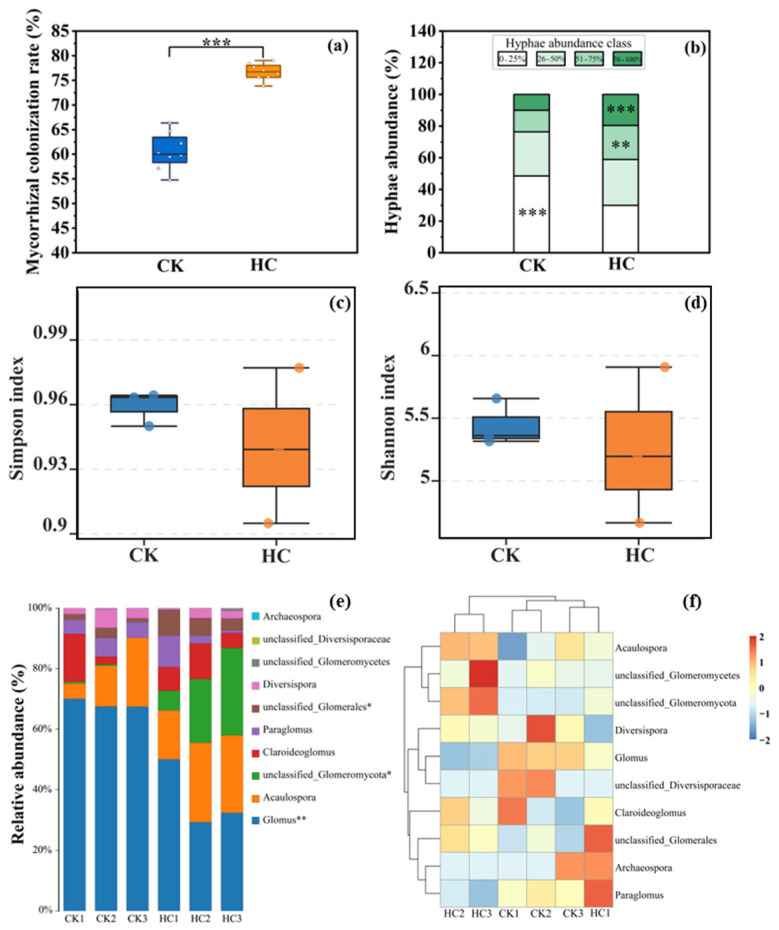
Mycorrhizal colonization rate (**a**), hyphae abundance (**b**), Simpson index (**c**), Shannon index (**d**), composition distribution and relative abundance (**e**), and the cluster heatmap of the species abundance at genus level of AMF (**f**) in the rhizosphere soil of *Sphagneticola trilobata* under different treatments. CK: population without herbicide application treatment; HC: population with herbicide application treatment. (* *p* < 0.05; ** *p* < 0.01; *** *p* < 0.001, Mean ± SE, *n* = 8).

The mycorrhizal colonization rate in the glyphosate-exposed field population was significantly higher than the control population (Figure 2a, *p* < 0.001). The hyphae abundance range in 0–25% was higher in the CK population compared with the HC population. While the hyphae abundance range in 50–75% and 75–100% were significantly higher in the HC population compared to the CK population (Figure 2b).

We found that there were no significant differences in the Simpson index and the Shannon index of the AMF community between CK and HC populations (Figure 2c,d). The AMF communities had the highest relative abundances for the genus *Glomus*. However, there were obviously differences between these two populations. There were significant differences between these two populations in the relative abundance of *Glomus*, unclassified-*Glomeromycota,* and unclassified *Glomerales* (Figure 2e). The species abundance clustering heatmap also showed that there were significant differences between CK and HC populations in the rhizosphere AMF community (Figure 2f).

### 3.3. Metabolomics Analysis of the Effects of AMF on S. trilobata

**Figure 3:**
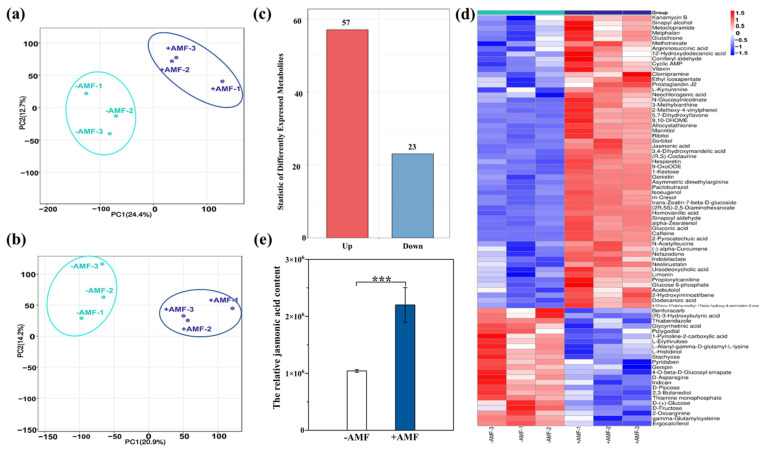
PCA scores of positive ion mode (**a**), PCA scores of negative ion mode (**b**), and jasmonic acid (**e**) relative content of *Sphagneticola trilobata* under different treatments. Statistical histogram (**c**) and heat map (**d**) of different expressed metabolites of *Sphagneticola trilobata* under different treatments. −AMF: without AMF inoculation; +AMF: with AMF inoculation (*** *p* < 0.001, Mean ± SE, *n* = 3).

As the application of herbicides caused significant differences in the AMF communities of *S. trilobata*, we conducted a non-targeted metabolome analysis using the plant samples inoculated with AMF to explore the metabolic mechanism. The principal component analysis (PCA) exhibited differences between with and without AMF inoculation along the first principal component (PC1) (Figure 3a,b). Notably, PC1 accounted for 24.4% and 20.9% of the total variance in the positive and negative ion modes, respectively.

Differential metabolites were screened from the sample-level substance list using the predefined *p* ≤ 0.05 and VIP ≥ 1 thresholds in statistical tests. Among them, VIP stands for Variable Importance in the Projection, and it is a core indicator used in the OPLS-DA model for filtering key variables. Generally, in the context of OPLS-DA, it specifically refers to the contribution of the first predictive principal component, so the full name is VIP score for the first predictive component. It is generally considered that variables with VIP values greater than 1 have statistical significance in distinguishing groups. These variables are potential biomarkers or key differentiators. In this experiment, 80 differential metabolites were identified from these two treatments, including 57 up-regulated and 23 down-regulated metabolites (Figure 3c,d). The metabolites were significantly different between these two treatments (Figure 3d). Especially, inoculating with AMF significantly increased the relative content of jasmonic acid (Figure 3e).

### 3.4. Effects of JA and AMF on the Growth of S. trilobata Under Herbicide Application

**Figure 4:**
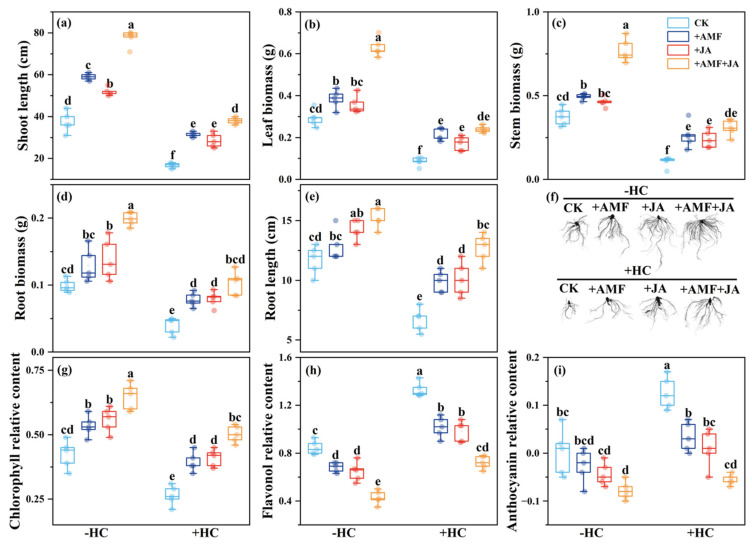
Stem length (**a**), leaf biomass (**b**), stem biomass (**c**), root morphology (**d**), root length (**e**), root biomass (**f**), chlorophyll relative content (**g**), flavonol relative content (**h**), and anthocyanin relative content (**i**) of *Sphagneticola trilobata* under different treatments. −HC: without herbicide application; +HC: with herbicide application; CK (Light blue): without AMF inoculation or methyl dihydrojasmonate application; +AMF (Dark blue), with AMF inoculation; +JA (Red), with methyl dihydrojasmonate application; +AMF + JA (Orange), with AMF inoculation and methyl dihydrojasmonate application. Different letters indicate significant differences in plants under different treatments (*p* < 0.05, Mean ± SE, *n* = 5).

In the absence of herbicide, both AMF inoculation and JA application significantly increased the stem length of *S. trilobata* (Figure 4a). Furthermore, +AMF +JA treatment remarkably increased plant stem length, leaf biomass, and stem biomass compared to their individual effects (Figure 4a–c). Herbicide application significantly reduced the stem length, leaf biomass, and stem biomass of *S. trilobata* (Figure 4a–c). Under herbicide stress, +AMF or +JA treatment significantly increased stem length, leaf biomass, and stem biomass. +AMF +JA treatment further significantly increased these growth traits, and also better than their individual effects (Figure 4a–c).

The root morphology of *S. trilobata* was changed by AMF inoculation, methyl dihydrojasmonate, and herbicide application (Figure 4d). Without herbicide, AMF inoculation did not significantly affect, whereas +JA treatment significantly increased root length or root biomass. Furthermore, +AMF +JA treatment significantly enhanced root length and biomass, and also increased their individual effects (Figure 4e,f). Herbicide application significantly reduced root length and root biomass. However, under herbicide stress, +AMF or +JA treatments significantly increased root length and root biomass. In addition, compared with their individual effects, the combined treatment significantly increased root length and root biomass under herbicide application (Figure 4e,f).

In the absence of herbicide, both +AMF and +JA significantly increased chlorophyll relative content and decreased flavonol relative content in *S. trilobata*, while anthocyanin relative content remained unchanged. Also, +AMF + JA treatment significantly increased chlorophyll and decreased flavonol relative content without affecting anthocyanin levels (Figure 4g–i). With herbicide application, chlorophyll relative content significantly decreased, whereas flavonol and anthocyanin relative contents significantly increased. Both +AMF and +JA treatments significantly increased chlorophyll relative content and decreased both flavonol and anthocyanin relative contents. Compared with their individual effects, +AMF +JA treatment significantly increased chlorophyll relative content while significantly decreasing flavonol and anthocyanin relative contents (Figure 4g–i).

## 4. Discussion

In this study, we found that invasive *S. trilobata* had greater herbicide tolerance than its native congener *S. calendulacea*. In the field survey, we found that herbicides could change AMF colonization and diversity in the *S. trilobata* population. AMF inoculation could significantly change the metabolites of *S. trilobata*, especially JA content. Lastly, we found that AMF symbiosis and JA application could enhance the herbicide resistance of *S. trilobata*; also, their combination effects had greater effects than their individual effects.

### 4.1. Role of AMF in Plant Resistance to Herbicides

In the field experiment, we found that herbicide significantly increased AMF colonization rate and changed AMF community in the invasive weed *S. trilobata* (Figure 2). Some recent reports have also suggested that herbicide stress can increase colonization rates [46,56]. This may be because plants facing moderate to low levels of chemical stress activate compensatory or defensive mechanisms [57,58]. In addition, microorganisms can metabolize herbicides to support their growth [59,60], which may release more nutrients into the soil microbial community and support higher microbial diversity, including AMF diversity [61]. To better absorb nutrients and water and resist the physiological stress caused by herbicides, plants may actively strengthen their symbiotic relationship with AMF [62,63,64], secreting more signaling substances (such as lonicerin) to attract AMF colonization and provide them with more energy [65,66].

On the other hand, the dominant AMF communities had changed in the herbicide application population (Figure 2). Among these, *Glomus* and unclassified-*Glomeromycota*, *Acaulospora*, and *Claroideoglomus* can form symbiotic associations with plant roots, significantly influencing the host’s nutrient absorption and especially stress tolerance [67,68,69,70], for example, *Glomus* can enhance the absorption of nutrients such as phosphorus, promote proline accumulation, and enhance plant’s tolerance to drought [71,72]; *Glomeromycota* can enhance plant’s resistance to biotic and abiotic stresses by activating its local and systemic defense mechanisms [73]; *Acaulospora* can improve wheat’s tolerance efficiency to alkaline stress [74]; *Claroideoglomus* enhance soybean’s symbiotic performance, increase nitrogenase activity, and also enhance the growth, phosphorus uptake, and phosphate transporter gene *Pht* expression of olive (*Olea europaea* L., Oleaceae) plantlets [75,76]. Under herbicide stress, *S. trilobata* may actively screen and recruit AMF partners that can help it degrade or tolerate herbicide toxicity or provide key nutrients more efficiently through chemical signals such as root exudates to ensure its survival [77,78]. Some studies have pointed out that invasive plants often develop higher stress adaptability through microbial interactions, giving them an advantage over native species [79].

### 4.2. Role of JA and Its Interaction with AMF in Plant Resistance to Herbicides

Study indicates that AMF are powerful biological allies in enhancing plants’ resistance to biotic and abiotic stresses [80]. Their mechanisms of action are comprehensive, including enhancing the survival, growth, and reproductive capacity of host plants under adverse conditions through synergistic mechanisms such as constructing extensive underground absorption networks, optimizing plant nutrient and water status, regulating ion balance and osmotic potential, activating antioxidant defense systems, and isolating and immobilizing toxic substances [81,82,83,84]. Studies have also shown that with symbiosis with AMF, host plants may undergo metabolic changes that confer advantages in the resistance to biotic and abiotic stress. Shan et al. [85] found that AMF primarily promote lateral root development and plant growth of apple (*Malus pumila* Mill., Rosaceae) by influencing glucose metabolism, fatty acid metabolism, and hormone metabolism.

Meanwhile, the invasive plant *S. trilobata* can increase the concentration of flavonoids in root exudates, strengthening its symbiosis with AMF, thereby enhancing its tolerance to environmental stress [86]. Our study also found that 80 differentially expressed metabolites had changed with AMF symbiosis (Figure 3). These altered secondary metabolites may play crucial roles in environmental adaptation and resistance to stress [87,88,89]. This study identified 35 differentially expressed metabolites with significantly increased relative abundance and large fold changes, including jasmonic acid (JA). Studies have shown that JA regulates antioxidant responses and activates the expression of defense genes to coordinate plant immune responses [90], thereby enhancing plant resistance to environmental stress. Research indicates that JA can also influence plants’ resistance to herbicides. For example, exogenous application of JA can regulate plant toxicity responses to mitigate the negative effects of the herbicide metamitron on tobacco (*Nicotiana tabacum* L., Solanaceae) [91]; Zhang et al. [92] noted that between wheat and neighboring ryegrass (*Lolium perenne* L., Poaceae), JA drives wheat to accelerate the release of hydroxamic acid into the environment and convert it into stable products, thereby activating the ryegrass antioxidant system or disrupting metabolic pathways, indirectly enhancing ryegrass’s adaptability to the stress of the herbicide imazethapyr, forming a synergistic defense mechanism between plants, and mitigating the stress effects of the herbicide on ryegrass. Other studies have also shown that JA hormone signaling may play an important role in the resistance of barnyard grass (*Echinochloa crusgalli* (L.) P.Beauv., Poaceae) to the stress of the herbicide quinoline acid [93]. Therefore, JA is one of the key factors in plants’ resistance to herbicide stress.

The results of this study also indicate that inoculation with AMF and application of jasmonate can alleviate the inhibitory effect of herbicide stress on the growth of *S. trilobata*. Glyphosate herbicides inhibit plant growth by blocking the shikimate pathway in plants, forming irreversible enzyme-inhibitor complexes, thereby inhibiting the synthesis of essential protein precursors and aromatic amino acids required for plant growth [94], leading to metabolic disorders, growth arrest, and ultimately death [95,96]. Concurrently, the accumulation of shikimate and its precursor compounds within plants leads to the accumulation of reactive oxygen species (ROS), thereby inducing oxidative damage in plants [97], a mechanism similar to that of certain pathogenic organisms infecting plants [98,99]. Mycorrhizal colonization can enhance plant disease resistance by inducing the synthesis of jasmonic acid and salicylic acid in plants [100] or by activating the plant antioxidant enzyme system (e.g., POD, PPO) and defense genes (e.g., MYC2) through the jasmonic acid pathway, thereby enhancing resistance to pathogens [101]. Therefore, in this study, when *S. trilobata* is subjected to herbicide stress, interaction with AMF can activate the defense system of *S. trilobata*, regulate plant-related physiological and signal transduction pathways, and likely increase endogenous hormone content and induce the production of stress-related secondary metabolites [102,103], thereby enhancing the tolerance of *S. trilobata* plants to herbicides.

On the other hand, this study indicates that JA and AMF exhibit synergistic effects in enhancing *S. trilobata*’s herbicide resistance against glyphosate-induced stress (Figure 4). These findings are consistent with previous studies, indicating that JA promotes symbiotic relationships and creates a favorable environment for AMF even under chemical stress [104], and highlight JA’s role in enhancing plant defense mechanisms and alleviating oxidative stress by triggering antioxidant responses (such as upregulating flavonoid synthesis) [105,106,107,108]. This synergistic effect may be attributed to JA promoting AMF colonization, while AMF helps maintain nutrient flow (particularly phosphorus, which is crucial for chlorophyll synthesis) and stress tolerance [109]. AMF helps maintain photosynthetic efficiency by improving nutrient absorption, enabling *S. trilobata* to better tolerate herbicide-induced damage. This also explains the significant increase in chlorophyll content observed when JA and AMF are applied together [110]. These findings have important implications for developing integrated weed management strategies that reduce reliance on chemical herbicides by combining JA and AMF.

### 4.3. Ecological Implications of AMF Symbiosis in Invasive Weed Resistance to Herbicide

This study reveals the important roles of AMF and their interaction with the plant hormone jasmonic acid in enabling invasive weeds to respond to herbicide exposure. Previous studies have also demonstrated that AMF and plant hormones can promote plant invasion in multiple ways [111]. For example, AMF can significantly enhance phosphorus uptake and growth rate in *Sporobolus alterniflorus* Loisel. P.M.Peterson & Saarela (Poaceae) under phosphorus-deficient conditions [109,112]. AMF can also enhance the environmental stress tolerance of invasive plants, including salt and drought stresses [113,114]. AMF symbiosis with ragweed (*Ambrosia artemisiifolia* L., Asteraceae) could contribute to its competition with other native plants [115]. Additionally, AMF can assist invasive plants in resource allocation and promote plant invasion [116,117]. On the other hand, AMF can also interact with plant hormones through signal interaction and functional complementarity to further enhance invasion [118,119].

Herbicides are currently the most effective chemical and are widely used in weed management [120]. However, in this study, we found that AMF symbiosis could regulate jasmonic acid to enhance the herbicide resistance of the invasive weed. The widespread use of herbicides also has led to the herbicide tolerance of plant species [121,122]. That might lead to greater herbicide applications, resulting in a vicious circle in weed management. Consequently, incorporating the interaction of JA and AMF into weed management strategies, such as by disrupting the AMF symbiosis with weed or blocking the pathway of JA synthesis, might improve the efficiency of herbicide application. Moreover, further exploration of the mechanisms of interactions of JA and AMF response to herbicides will provide deeper insights into the management of invasive species. Disrupting the close relationship between plants and mycorrhiza symbiosis, and also decreasing plant JA content, might improve weeding efficiency, which might provide a new potential idea for weed control.

In summary, we found that herbicide application could significantly affect the AMF diversity of the invasive weed *S. trilobata* and also that AMF symbiosis could enhance JA content. We also verified that the interaction of JA and AMF symbiosis could enhance the resistance to herbicides of the invasive weed. The scientific novelty of this study lies in the new theoretical basis and sustainable control strategies for invasive plant management, as well as important references for research on plant-microbe interactions and stress resistance mechanisms. We are looking forward to further studies that reveal the key microbial taxa, functional genes, and signaling pathways, and elucidate the molecular and physiological ecological mechanisms that drive plant invasion and enhance stress resistance. At the same time, we should focus on how these interaction mechanisms respond dynamically in the context of climate change (such as drought, saline-alkali, and other compound stresses), so as to lay a foundation for predicting the evolution trend of invasive plants in changing environments and providing forward prevention and control strategies.

## Data Availability

The original contributions presented in this study are included in the article/Supplementary Material. Further inquiries can be directed to the corresponding authors.

## Supplementary Materials

The following supporting information can be downloaded at: https://www.mdpi.com/article/10.3390/microorganisms13122817/s1. Figure S1: The population of *Sphagneticola trilobata* without herbicide application (a) and the new-born shoots of *Sphagneticola trilobata* after glyphosate application (b).
