# Supplementary material for: The Synergistic Effects of Jasmonic Acid and Arbuscular Mycorrhizal Fungi in Enhancing the Herbicide Resistance of an Invasive Weed Sphagneticola trilobata
Source: Microorganisms. 2025 Dec 10;13(12):2817. doi: 10.3390/microorganisms13122817 (PMC12735793; doi:10.3390/microorganisms13122817)
Supplement: Supplementary file 1 [file microorganisms-13-02817-s001.zip › microorganisms-3974746-supplementary.pdf]

microorganisms

MDPI

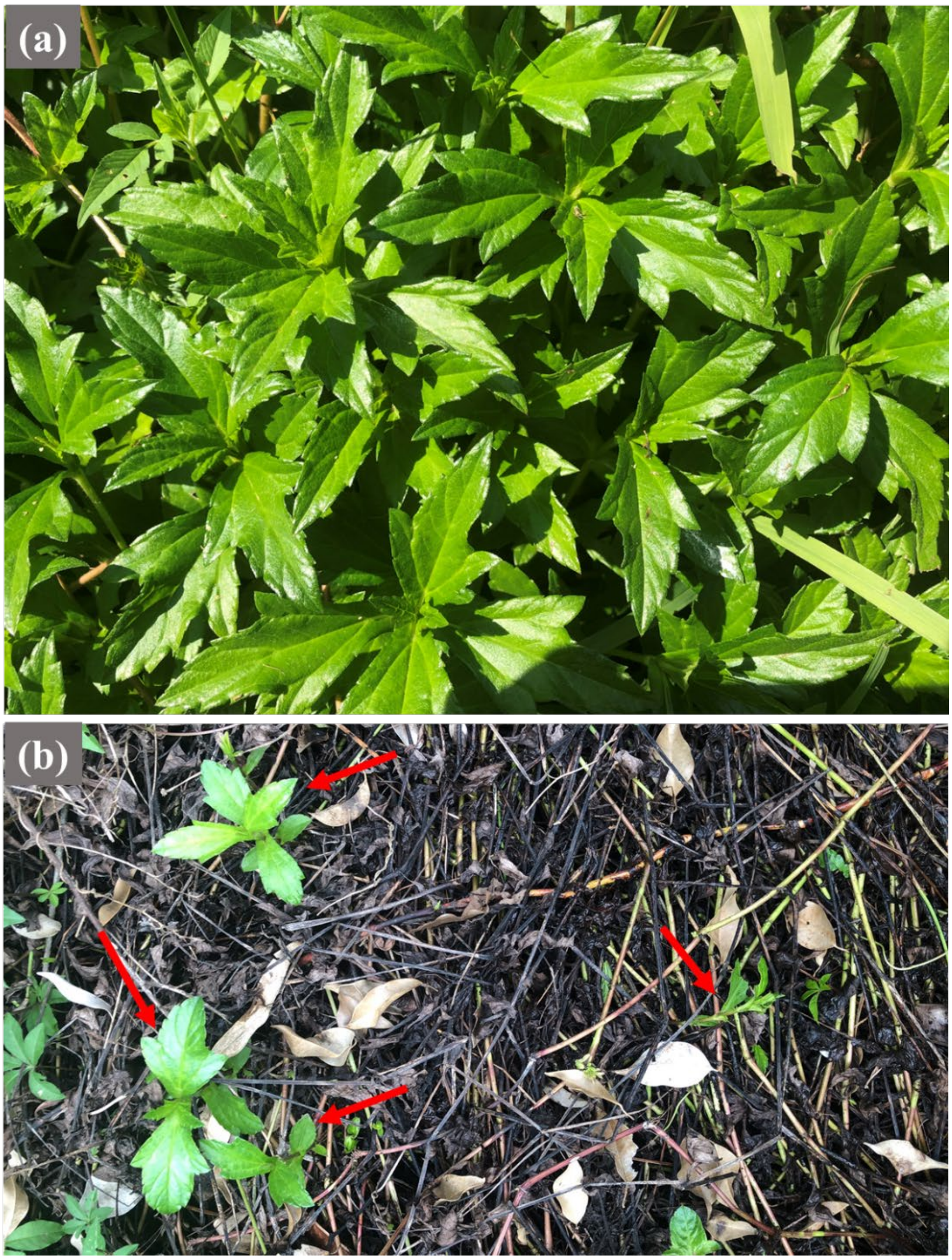

Figure S1 The population of *Sphagneticola trilobata* without herbicide application (a) and the new-born shoots of *Sphagneticola trilobata* after glyphosate application (b).

 https://doi.org/10.3390/microorganisms13122817
